# Left Ventricular Contraction Duration Is the Most Powerful Predictor of Cardiac Events in LQTS: A Systematic Review and Meta-Analysis

**DOI:** 10.3390/jcm9092820

**Published:** 2020-08-31

**Authors:** Mena Abdelsayed, Ibadete Bytyçi, Annika Rydberg, Michael Y. Henein

**Affiliations:** 1 Institute of Public Health and Clinical Medicine, Umeå University, 90187 Umeå, Sweden; menaabdelsayed1993@gmail.com (M.A.); i.bytyci@hotmail.com (I.B.); 2 Universi College, Bardhosh, 10000 Prishtina, Kosovo; 3 Department of Clinical Sciences, Pediatrics, Umeå University, 90187 Umeå, Sweden; annika.rydberg@pediatri.umu.se; 4 Molecular and Clinical Sciences Research Institute, St George University London, SW17 0QT, UK; 5 Institute of Fluid Dynamics, Brunel University, London UB8 3PH, UK

**Keywords:** contraction duration, long-QT syndrome, electromechanical window, asymptomatic, symptomatic

## Abstract

Background: Long-QT syndrome (LQTS) is primarily an electrical disorder characterized by a prolonged myocardial action potential. The delay in cardiac repolarization leads to electromechanical (EM) abnormalities, which adds a diagnostic value for LQTS. Prolonged left ventricular (LV) contraction was identified as a potential risk for arrhythmia. The aim of this meta-analysis was to assess the best predictor of all EM parameters for cardiac events (CEs) in LQTS patients. Methods: We systematically searched all electronic databases up to March 2020, to select studies that assessed the relationship between echocardiographic indices—contraction duration (CD), mechanical dispersion (MD), QRS onset to peak systolic strain (QAoC), and the EM window (EMW); and electrical indices— corrected QT interval (QT_C)_, QT_C_ dispersion, RR interval in relation to CEs in LQTS. This meta-analysis included a total of 1041 patients and 373 controls recruited from 12 studies. Results: The meta-analysis showed that LQTS patients had electrical and mechanical abnormalities as compared to controls—QT_C_, WMD 72.8; QT_C_ dispersion, WMD 31.7; RR interval, WMD 91.5; CD, WMD 49.2; MD, WMD 15.9; QAoC, WMD 27.8; and EMW, WMD −62.4. These mechanical abnormalities were more profound in symptomatic compared to asymptomatic patients in whom disturbances were already manifest, compared to controls. A CD ≥430 ms had a summary sensitivity (SS) of 71%, specificity of 84%, and diagnostic odds ratio (DOR) >19.5 in predicting CEs. EMW and QT_C_ had a lower accuracy. Conclusions: LQTS is associated with pronounced EM abnormalities, particularly prolonged LV myocardial CD, which is profound in symptomatic patients. These findings highlight the significant role of EM indices like CD in managing LQTS patients.

## 1. Introduction

Almost three decades have passed since M-mode echocardiography confirmed that Long-QT syndrome (LQTS) is associated with electromechanical (EM) abnormalities [1]. In the past, LQTS was characterized purely as an electrical abnormality of the myocardium that results in cardiac action potential (AP) prolongation. LQTS patients with mechanical dysfunction have impairments in both systolic and diastolic functions, as a result of prolonged QT_C_ [2]. The prolonged QT duration in LQTS increases the susceptibility for exacerbated heterogeneity in repolarization between myocardial cells. Repolarization timing between myocardial cells differs as AP duration is longest in endocardial Purkinje cells and subendocardial to midmyocardial cells (M cells), as compared to epicardial cells [3]. The difference in repolarization timing is further exacerbated in LQTS leading to an electrical dispersion in the myocardium, underlies extrasystoles, and other ectopic ventricular beats that potentially degenerate into polymorphic ventricular tachycardia [4]. A mechanical dispersion in the myocardium ensues as a result of this electrical heterogeneity in LQTS patients [5]. Strain-imaging proved that a degree of correlation exists between the AP duration and myocardial contraction duration [5].

Symptomatic LQTS patients have an increased risk of experiencing syncope or cardiac events like torsades de pointes or polymorphic ventricular tachycardia, which culminate in cardiac arrest [6]. Studies proved that electromechanical dysfunction is worse in symptomatic vs. asymptomatic LQTS patients [7]. Several groups confirmed systolic and diastolic dysfunction in LQTS, using M-mode or Tissue Doppler (TD) echocardiography [7]. Various echocardiographic indices measured by Doppler and strain echocardiography were analyzed in studies linking mechanical to electrical abnormalities in LQTS [5,7]—QT_C_, QT_C_ dispersion, RR interval, contraction duration (CD), mechanical dispersion (MD), EM window (EMW), and onset QRS to peak systolic strain (QAoC). There are more powerful echocardiographic indices besides the traditional that are important to analyze to understand risk stratification in LQTS patients. Electrocardiographic indices, along with other traditional measures, such as occurrence of syncope, genotype, and gender, are in fact limited in detecting left ventricular (LV) heterogeneity in LQTS patients [8]. An increase in CD is most evidently linked to prolonged QT_C_ in LQTS patients [9]. Other mechanical parameters are correlated with QT_C_ [2]. An increased CD can lead to diastolic dysfunction that decrease LV filling time [2]. Despite these electromechanical abnormalities, LQTS patients display normal LV function [4,10]. Of all the electromechanical indices measured from LQTS patients, this meta-analysis study aimed to assess the index that is best at predicting cardiac events in LQTS as a means of risk stratification.

## 2. Materials and Methods

We followed the 2009 guidelines preferred reporting items for systematic reviews and meta-analysis (PRISMA) statement [11], amendment to the Quality of Reporting of Meta-analyses (QUOROM) statement [12]. Due to the study design (meta-analysis), neither Institutional Review Board (IRB) approval nor patient informed consent was needed.

### 2.1. Search Strategy

We systematically searched PubMed-Medline, EMBASE, Scopus, Google Scholar, the Cochrane Central Registry of Controlled Trials and ClinicalTrial.gov, up to March 2020, using the following key words: “Long QT syndrome” OR “LQTS” OR “Long QT interval” AND “Left ventricle” OR “Left ventricle function” OR “Myocardial deformation” OR “Left ventricle strain” OR “Mechanical dispersion” OR “Contraction duration” AND “Echocardiography”. 

Additional searches for potential trials included the references of review articles on that issue, and the abstracts from selected congresses—scientific sessions of the European Society of Cardiology (ESC), the American Heart Association (AHA), American College of Cardiology (ACC), and European Heart Rhythm Association (EHRA). The wild-card term ‘‘*’’ was used to increase the sensitivity of the search strategy. The literature search was limited to articles published in English and to studies in humans. Two reviewers (MA and IB) independently evaluated each article. No filters were applied. Disagreements were resolved by discussion with a third party (MYH).

### 2.2. Study Selection

***Inclusion criteria*** in the meta-analysis were—(a) data for patients with Long-QT syndrome and control group; (b) reporting EM abnormalities; and (c) enrolled human subject.

***Exclusion criteria*** were: (a) insufficient statistical data to compare two groups; (b) only one group of treatments (symptomatic or asymptomatic); (c) non-human subjects; and (d) articles not published in English.

### 2.3. Outcome Variables

Key clinical end points were the relationship between EM abnormalities and cardiac events (CEs). Main outcome measures were echocardiographic indices—contraction duration (CD) measured from the onset of the R wave to the end of post-ejection velocity, mechanical dispersion (MD) measured from the standard deviation of contraction duration, aortic valve closure time (QAoC) measured from the difference between QRS onset to peak systolic strain, and the EM window (EMW) measured from the time difference between the end of electrical systole (end of QT interval) and the completion of mechanical systole (onset of aortic valve closure); and electrical indices—QT_C_, QT_C_ dispersion, and RR interval. CEs was defined as documented arrhythmia, syncope, or cardiac arrest.

### 2.4. Data Extraction

Eligible studies were reviewed and the following data were abstracted—(1) first author’s name; (2) year of publication; (3) study design; (4) data on two arms; LQT and control group; (5) measures of electrical and echocardiographic abnormalities; (6) baseline characteristics of the patients; and (7) age and gender of study participants.

### 2.5. Quality Assessment

Assessment of risk of bias in the cohort studies was evaluated by the same investigators, using the Newcastle-Ottawa Scale (NOS). Three domains were evaluated with the following items: (A) Selection—(1) representativeness of the exposed cohort, (2) selection of the non-exposed cohort, (3) ascertainment of exposure and (4) demonstration that outcome of interest was not present at start of study. (B). Comparability of exposed and non-exposed. and (C) Exposure— (1) assessment of outcome, (2) follow-up long enough for outcomes to occur, and (3) adequacy of the follow-up of cohorts. The risk of bias in each study was judged to be “good”, “fair”, or “poor” [13].

### 2.6. Statistical Analysis

The meta-analysis was conducted using statistical analysis, performed using the RevMan (Review Manager (RevMan) Version 5.1, The Cochrane Collaboration, Copenhagen, Denmark), with two-tailed *p* < 0.05 considered to be significant. Relative risk (RR) ratios with 95% confidence interval (CI) are presented as summary statistics, whereas for continuous variables, weighted mean differences (WMD) and 95% CI were used. The baseline characteristics are reported as median and range. Mean and standard deviation (SD) values were estimated using the method described by Hozo et al. [14]. Analysis is presented in forest plots, the standard way for illustrating the results of individual studies and meta-analysis. To evaluate the relationship between LV EM abnormalities and CEs, we performed hierarchical summary receiver operating characteristic (ROC) analysis using the Rutter and Gatsonis model [15]. Summary sensitivity and specificity with 95% CI for individual studies based on true positive (TP), true negative (TN), false positive (FP), and false negative (FN) were computed using the diagnostic random-effects model [16]. Summary point from the hierarchical ROC analysis was then used to calculate the positive likelihood ratio (LR+), negative likelihood ratio (LR–), positive predictive value (PPV), negative predictive value (NPV), and diagnostic odds ratio (DOR). In studies that did not provide optimal cutoffs, we created the ROC curve and identified the optimal cutoff as the point on the ROC curve closest to 0.1 on the x-y coordinate. The RevMan was used for statistical analysis including graphic presentations of forest plots of sensitivity and specificity, as well as hierarchical summary ROC curves.

Heterogeneity between studies was assessed using Cochrane Q test and *I*^2^ index. As a guide, *I*^2^ < 25% indicated low, 25–50% moderate, and >50% high heterogeneity [17]. To assess the additive (between-study) component of variance, the reduced maximum likelihood method (*tau*^2^) incorporated the occurrence of residual heterogeneity into the analysis [8]. Publication bias was assessed using visual inspections of funnel plots and Egger’s test.

## 3. Results

### 3.1. Search Results and Trial Flow

Of 1099 articles identified in the initial search, 545 were screened as potentially relevant. After careful assessment of 34 full articles, only 12 articles with a total 1041 patients, and 373 controls were finally included [2,4,5,7,8,18,19,20,21,22,23,24] (Figure S1).

### 3.2. Characteristics of Included Studies

Of the 1041 included patients and 373 controls from 12 cohort studies (Table 1), 415 were symptomatic and 554 were asymptomatic; however, 72 patients were not classified based on symptoms [7,20]. LQTS patients had a similar age (33.1 ± 13.8 vs. 33.3 ± 11.9 years, *p* = 0.73) and a female percentage (58.1% vs. 54.2%, *p* = 0.09), compared to control. Symptomatic patients had a higher female prevalence (71.5 vs. 61.4%, *p* = 0.03) compared to asymptomatic patients, despite a minimal age difference between the two groups (27.7 ± 12.7 vs. 31.2 ± 12.9 years, *p* = 0.07, Table S1).

### 3.3. Electrical Abnormalities in LQTS vs. Controls

Electrocardiographic indices were available from 10/12 studies analyzed in the meta-analysis [2,4,5,7,8,20,21,22,23,24]. Patients had a significantly prolonged QT_C_ duration, with a weighted mean difference (WMD) 72.8 [95% CI 60.8 to 84.1, I^2^ = 64%, *p* < 0.00001]); greater QT_C_ dispersion, WMD 31.7 (95% CI 11.8 to 51.7, I^2^ = 61%, *p* < 0.002); and prolonged RR interval, WMD 91.6 (95% CI 61.4 to 121.8, I^2^ = 3%, *p* < 0.00001) compared to the controls (Figure S2a–c).

### 3.4. LV Mechanical Abnormalities in LQTS Patients vs. Controls

**Figure 1:**
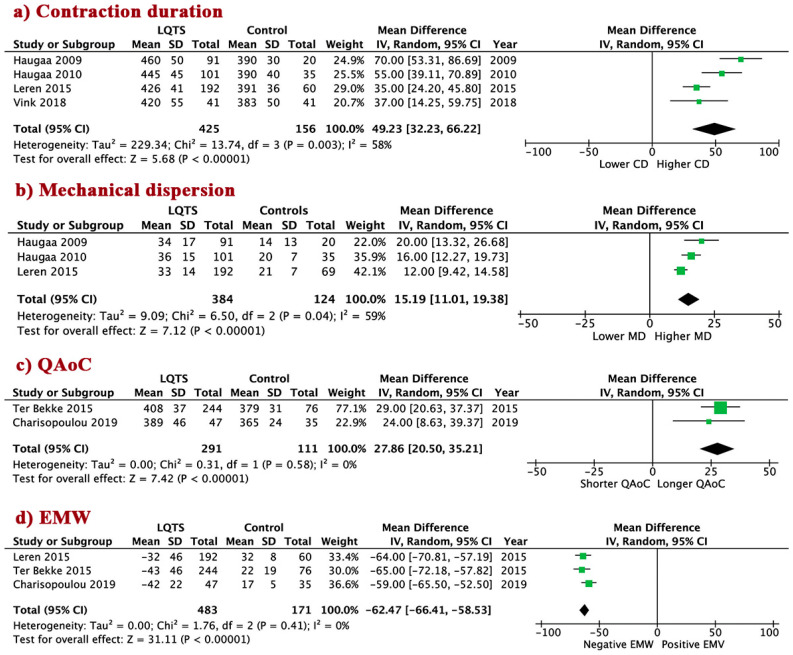
Mechanical abnormalities in long-QT syndrome (LQTS) patients vs. control. (**a**) Conctraction duration; (**b**) Mechanical dispersion; (**c**) QAoC; (**d**) EMW. LQTS: long QT syndrome; QAoC: QRS onset to peak systolic strain, EMW: electro-mechanical window; CD: contraction duration; MD: mechanical dispersion.

To assess LV mechanical abnormalities in LQTS patients, we measured four echocardiographic indices—CD, MD, QAoC, and EMW. Six of the twelve studies analyzed [2,5,18,21,23,24], unevenly measured these indices from a total of 716 LQTS patients. Compared to the controls, LQTS patients had prolonged CD, WMD 49.2 (95% CI 32.2 to 66.2, I^2^ = 58%, *p* < 0.00001); higher MD, WMD 15.2 (95% CI 11.0 to 19.4, I^2^ = 59%, *p* < 0.00001); prolonged QAoC, WMD 27.9 (95% CI 20.5 to 35.2, I^2^ = 0%, *p* < 0.00001); and more negative EMW, WMD −62.5 (95% CI −66.4 to −58.5, I^2^ = 0%, *p* < 0.00001) (Figure 1a–d).

LV global longitudinal strain (LV GLS) was also reduced in LQTS patients compared to controls, WMD 1.07 (95% CI 0.62 to 1.53, I^2^ = 0%, *p* < 0.00001, Figure S3b). However, LV ejection fraction (LVEF) was preserved in patients and was not significantly different from the controls, WMD 0.33 (95% CI −0.50 to 1.16, I^2^ = 0%, *p* = 0.43, Figure S3a). In contrast, diastolic function was compromised, as evidenced by lower E/A ratio, WMD −0.14 (95% CI −0.23 to −0.05, I^2^ = 0%, *p* < 0.002); longer E deceleration time, WMD 43.40 (95% CI 19.6 to 67.2, I^2^ = 67%, *p* < 0.0004); and prolonged isovolumic relaxation time (IVRT), WMD 7.12 (95% CI 0.71 to 13.52, I^2^ = 57%, *p* < 0.03) in LQTS patients, compared to controls (Figure S4a–c).

### 3.5. LV Mechanical Abnormalities in Asymptomatic vs. Symptomatic LQTS Patients

**Figure 2:**
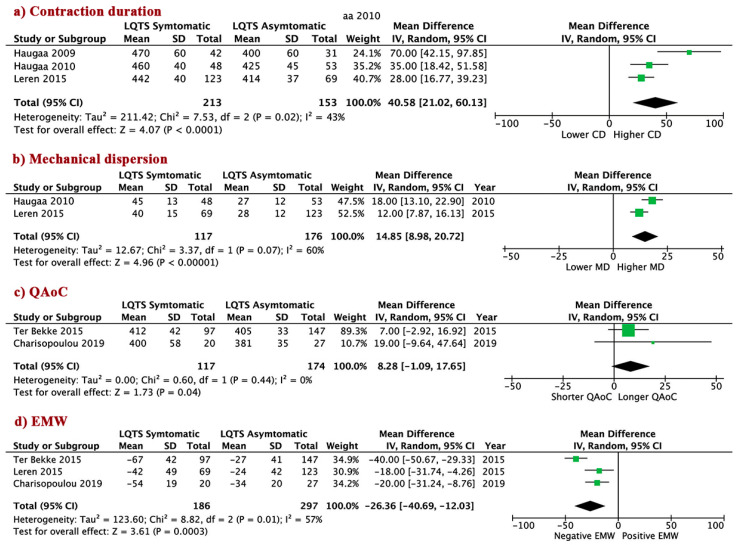
Mechanical abnormalities in LQTS patients—comparison of symptomatic vs. asymptomatic patients. (**a**) Conctraction duration; (**b**) Mechanical dispersion; (**c**) QAoC; (**d**) EMW. LQTS: long QT syndrome; QAoC: QRS onset to peak systolic strain, EMW: electro-mechanical window; CD: contraction duration; MD: mechanical dispersion

Of the total 1041 included patients, comparison of mechanical abnormalities indices between symptomatic vs. asymptomatic were available in 5 papers (276 symptomatic and 381 asymptomatic patients). Similar observations were seen when comparing symptomatic to asymptomatic LQTS patients. Symptomatic patients had longer CD, WMD 40.6 (95% CI 21.0 to 60.1, I^2^ = 43%, *p* < 0.0001); higher MD, WMD 14.9 [95% CI 9.0 to 20.7, I^2^ = 60%, *p* < 0.00001]; prolonged QAoC, WMD 8.3 (95% CI −1.1 to 17.7, I^2^ = 0%, *p* = 0.04); and profoundly negative EMW, WMD −26.4 (95% CI −40.7 to −12.0, I^2^ = 57%, *p* < 0.0003), compared to asymptomatic patients (Figure 2a–d). Although the WMD is not as large, QT_C_ corroborates the CD difference between symptomatic and asymptomatic patients (Figure S5).

### 3.6. LV Mechanical Abnormalities in Asymptomatic LQTS Patients vs. Controls

**Figure 3:**
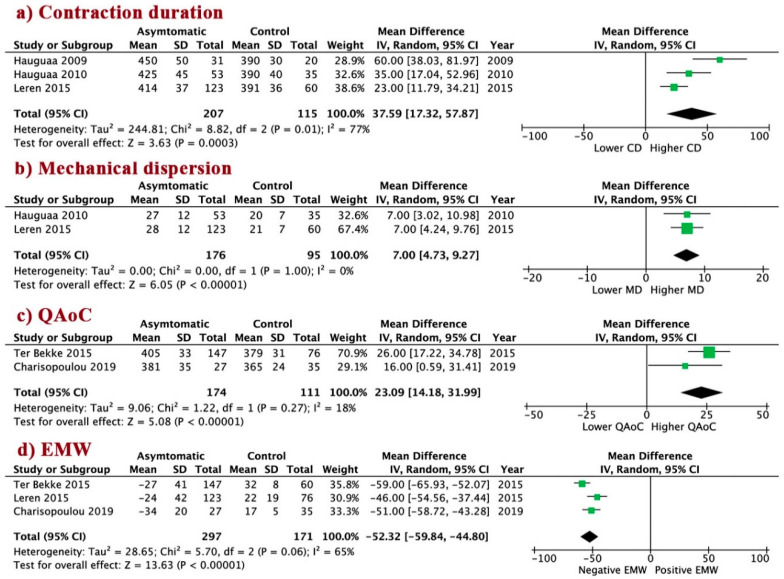
Mechanical abnormalities in asymptomatic patients vs. control. (**a**) Conctraction duration; (**b**) Mechanical dispersion; (**c**) QAoC; (**d**) EMW. LQTS: long QT syndrome; QAoC: QRS onset to peak systolic strain, EMW: electro-mechanical window; CD: contraction duration; MD: mechanical dispersion.

Compared to controls, the asymptomatic patients had significantly longer CD, WMD 37.6 (95% CI 17.3 to 57.9, I^2^ = 77%, *p* = 0.0003); larger MD, WMD 7.0 (95% CI 4.7 to 9.3, I^2^ = 0%, *p* < 0.00001); prolonged QAoC, WMD 23.1 (95% CI 14.2 to 32.0, I^2^ = 18%, *p* < 0.00001); and more negative EMW, WMD −52.3 (95% CI −59.8 to −44.8, I^2^ = 65%, *p* < 0.00001, Figure 3a–d), as compared to the control subjects.

### 3.7. EM Abnormalities in LQT1 vs. LQT2

Differences in EM properties between 268 LQT1 and 132 LQT2 patients were available in 4 papers. LQT1 and LQT2 patients had a similarly prolonged QT_C_ duration, WMD 3.33 (95% CI −2.11 to 8.77, I^2^ = 0%, *p* = 0.23, Figure S6a). No significant differences were found in the echocardiographic indices between both LQT1 and LQT2 patients—CD, WMD −5.37 (95% CI −31.7 to 20.9, I^2^ = 70%, *p* = 0.69); MD, WMD −2.94 (95% CI −7.71 to 1.84, I^2^ = 38%, *p* = 0.23); EMW, WMD 5.16 (95% CI −5.30 to 15.6, I^2^ = 0%, *p* = 0.33); and QAoC, WMD 6.57 (95% CI −9.77 to 22.9, I^2^ = 0%, *p* = 0.43, Figure S6b–e).

### 3.8. Electrical and Mechanical Predictors of Cardiac Events in LQTS Patients

**Figure 4:**
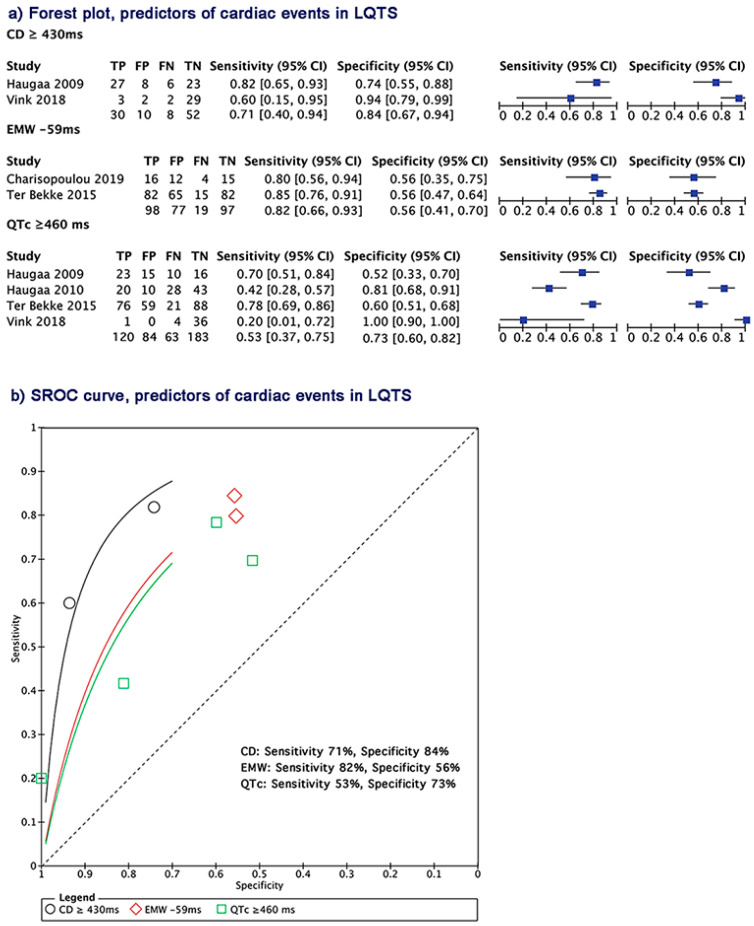
The Summary receiver operating characteristics (SROC) curve comparing CD, EMW, and QT_C_ duration in predicting CE in LQTS. CD: contraction duration; EMW: electromechanical window; QT_C_: corrected QT interval.

The predictors of CEs were present in 5 papers with 203 patients with CEs vs. 289 without CEs [5,18,21,23,24]. To determine the best predictor of CEs in LQTS patients, we used a hierarchical summary ROC analysis, which proved that the contraction duration was the strongest index compared to EMW and QT_C_. A cutoff of CD ≥ 430 ms predicted CE with a summary sensitivity of 71%, a summary specificity of 84%, accuracy of 83%, and a higher DOR > 19.5; EMW = −59 ms, with a summary sensitivity of 82%, summary specificity of 56%, accuracy of 67%, and a DOR > 7.47; and QT_C_ ≥ 460 ms with a summary sensitivity of 53%, summary specificity of 73%, accuracy of 68%, and DOR > 4.14 (Figure 4, Table S2).

### 3.9. Risk of Bias Assessment

Most cohorts had good quality, where approximately 20% had fair quality. There was also no evidence for publication bias, as evaluated by the Egger’s test.

## 4. Discussion

**Findings:** The findings of this meta-analysis could be summarized as follows—(1) LQTS patients had significant cardiac electromechanical abnormalities, irrespective of their genotype, LQT1 and LQT2, compared to control; (2) LQTS patients had preserved LVEF but reduced global longitudinal strain and compromised LV diastolic function; (3) mechanical abnormalities were worse in symptomatic compared to asymptomatic patients; (4) asymptomatic patients themselves had pronounced mechanical abnormalities compared to controls; and (5) a cutoff of CD ≥ 430 ms was more accurate in predicting cardiac events in LQTS patients compared to EMW = −47 ms and QT_C_ duration ≥ 460 ms.

**Data Interpretation:** LQTS was characterized as a pure electrical disorder for more than six decades [25], which limited our understanding of the underlying potential mechanisms of the disease in symptomatic patients. Over the last four decades, cardiac imaging techniques rapidly developed, with high precision in measuring cardiac cycle events in milliseconds. Doppler echocardiography was at the forefront of these advancements. Even the earliest generation of echocardiographic devices could report LV mechanical dysfunction associated with conduction abnormalities, e.g., left bundle branch block, as described by Edler [26]. Recently, echocardiographic speckle tracking technology was developed to assess myocardial deformation [27,28].

The aim of this meta-analysis was to ascertain LV mechanical abnormalities in LQTS, with an emphasis on symptomatic patients, to identify the strongest cardiac index that predicted occurrence of cardiac events. Despite a normal LV size and ejection fraction, the GLS, which reflects subendocardial function, was significantly reduced, a result that was consistent with previous findings [2]. The subendocardial layer might, therefore, have a selective role in LQTS pathophysiology, since it encompasses the purkinje fiber conduction system. Such disturbances have their clear impact on LV diastolic function, as we and others [29,30] previously showed in patients with coronary artery disease and heart failure, and preserved ejection fraction (HFpEF) [31]. Furthermore, our analysis showed that LV CD was significantly prolonged and EMW was abnormally negative, especially in symptomatic patients. However, CD was the strongest predictor of clinical events, even when compared with the conventionally reported prolonged QT_C_ in LQTS patients. These findings were closely interrelated, in a similar manner, to electromechanical disturbances in other conditions [32]. Increased CD prolonged the isovolumic relaxation time (IVRT) and delayed mitral valve opening, the product of which yielded a negative EMW, which resulted in a lowered E/A ratio (as reported) [2,9,33]. Consequently, early (E) diastolic LV filling volume was reduced, and so was the stroke volume and cardiac output, which might be ameliorated by beta blockers in symptomatic LQTS patients [34]. In addition, the relationship between electrical dysfunction and mechanical abnormalities were reported in other conditions like heart failure, cardiac resynchronization therapy (CRT), absent septal q wave, myocardial infarction, etc. [35,36,37,38,39,40] A possible explanation for this mechanism was that low stroke volumes might provoke arrhythmias in LQTS patients. Similar abnormalities exist in asymptomatic LQTS patients; however, with a severity less enough to cause symptoms. Additionally, the nature and extent of these abnormalities did not seem to be different between LQT1 and LQT2 subtypes.

**Mechanical and Molecular Mechanisms:** Mechanical dysfunction proved to be a pivotal marker for risk stratification in LQTS patients. From a pathophysiological perspective, the QT_C_ might not be an accurate index for measuring the risk of CE in LQTS patients compared to CD. The difference could be ascribed to the ability of the echocardiogram to encompass both high temporal and spatial resolutions, compared to a standard 12-lead ECG that only provides a measure of cardiac electric activity with high temporal resolution. Echocardiograms have a spatial resolution of more than 100 μm and a temporal resolution greater than 600 frames/s [41,42]. Different configurations like M-mode imaging has a high temporal resolution (>1000 frames s−1) [43], which allows for the visualization of rapid valve and ventricular wall movements. The standard 12-lead ECG, however, has a low resolution, compared to the echocardiogram [44]. The benefit of accounting for spatial resolution with cardiac indices is the ability to detect abnormalities in longitudinal and circumferential fibers of the ventricular wall. The sub-endocardium mainly consists of longitudinal fibers, oriented to 80° with respect to the circumferential fibers, which are condensed heavily in the basal and mid-cavity region [45,46]. The delay in electrical conduction between longitudinal and circumferential layers might generate an accentuated lag in depolarization and hence contraction in LQTS patients. Studies showed that CD is longer in longitudinal compared to circumferential fibers, resulting in a significant transmural mechanical dispersion in symptomatic patients [18]. This effect might be attributed to the presence of subendocardial Purkinje fibers, which have a significantly longer AP duration compared to the mid-myocardium [3]. De Ferrari et al. showed that prolonged CD in LQTS patients could be abolished by verapamil, suggesting a role played by L-type calcium current in the EM abnormalities [10]. Calcium, the excitation–contraction coupling ion, can both underlie electrical and mechanical disturbances. Due to a decrease in repolarization reserve in LQTS, the depolarization reserve prevails during the late phases of the action potential, which are specifically mediated by calcium ions. This compensatory mechanism might explain the preserved LVEF in LQTS patients, despite the presence of systolic and diastolic dysfunction. The depolarization wave mediated by calcium release in the myocardium of LQTS patients can differ, depending on the spatial region, yielding spatio-temporal heterogeneity within the wall of the myocardium [47]. Symptomatic patients might have a CD that exceeds that of aortic valve closure, signifying delayed contraction, despite the normal onset of ventricular diastole [47].

**Clinical implications:** LQTS is not a simple electrical abnormality but is associated with significant mechanical and electromechanical disturbances. These abnormalities have a profound impact on the overall cardiac performance in the form of compromised stroke volume, which could be an underlying trigger for arrhythmias. Left ventricular prolonged CD is the strongest predictor of clinical events in LQTS. Thus, it must be routinely measured in patients, even in the absence of symptoms. Adopting this suggestion could play an important role in optimum clinical management of LQTS patients.

**Study Limitations:** In this meta-analysis, little attention was given to EM abnormalities in genotype-specific LQTS patients, LQT1 and LQT2, due to the scarcity of the data. Our results showed that there were no differences between LQT1 and LQT2 patients with respect to the electrocardiographic and echocardiographic indices analyzed. The QT_C_ was shown to be inadequate in risk stratification in LQTS mutation carriers older than 40 years old [48]. LQTS genotypic differences ultimately determine a patient’s propensity to experience arrhythmic episodes during different physiological states, such as rest or exercise [49]. This factor might be another potential reason why we could not differentiate between latent EM abnormalities, since there is a lack of data on the effects of exercise, sleep, or other physiological/environmental triggers on EM parameters in LQT1 and LQT2 patients. Despite including the 12 studies included in this meta-analysis, a large variety existed in the parameters measured between studies; thus, only 2–4 studies were used in the calculation of the electro-mechanical parameters analyzed. Another potential limitation was the fact that we did not perform the electrocardiographic or echocardiographic measurements; however, we trust the analysis made by the authors of the 12 studies included in this meta-analysis. Likewise, we relied on the published data based on the statistics undertaken by the individual authors of those studies.

## 5. Conclusions

These findings seem to highlight that LQTS is associated with pronounced electromechanical abnormalities, particularly the prolonged LV myocardial contraction duration, which is profound in symptomatic patients, irrespective of their type. These findings highlight the significant role of such measurements, particularly LV contraction duration, in managing LQTS patients.

## Figures and Tables

**Table 1:** Main characteristics of studies included in the study.

Study (Year)	Study Design	Long QT Mutation	Inclusion Criteria	Exclusion Criteria	Electrical Abnormalities	Echocardiographic Abnormalities	Type of Long QT
Priori et al., 1994	Observational	Y—RWS	RWS Patients	Follow-up patients after institution of therapy of at least 1 year	QT, QT_C_, RR,	NR	RWS
					relative QT dispersion,		
					relative QT_C_ dispersion		
Nakayama et al., 1998	Observational—Prospective	NR	NR	NR	QT, QT_C_, QT	ThT	NR
					dispersion		
Savoye et al., 2003	Observational	NR	Screened after identifying symptomatic LQTS patients in family	Age ≥ 16, patients with pacemaker, valvular or myocardial disease	RR, QT_C_	LVDd, EF, E/A	LQT1
							LQT2
Leren et al., 2015	Cross-sectional	Y—DM and SM	LQTS mutation-positive subjects were included from our outpatient clinic.	Concomitant cardiac disease of other origin.	QT_C_, HR	EF%, EDV, GLS, MD, CD, EMW, e’, E deceleration time, IVRT, LAVI, E/A, E/e’	LQT1
							LQT2
				Patients with a diagnosis of hypertension or taking antihypertensive medication or with diabetes mellitus were excluded from diastolic measurements.			LQT3
			Only subjects with a pathogenic mutation were included.				
Haugaa et al., 2009	Observational	Y—SM and DM	Patients with previous cardiac	NR	QT_C_, RR	EF, CD by velocity, CD by strain, time to aortic valve closure, peak ejection velocity, PEV, Onset E’ wave, E/, E deceleration time	A and S
Haugaa et al., 2010	Observational	Y—Hom and Het	Genotyped patients	Asymptomatic patients younger than 18 years old	HR, QT_C_, QT_C_ dispersion	EF, Global strain, CD longitudinal, CD circumferential, MD longitudinal, MD circumferential	A and S;
							LQT1
							LQT2
							LQT3
							LQT5
Haugaa et al., 2013	Observational—Retrospective Study	Y	Patients evaluated in Mayo’s LQT Clinic between August 1998 till December 2008	24 patients who did not accept to participate	HR, QT_C_,	EF, Left atrial volume indexed, E, e/, E/e’, E deceleration time	A and S;
							LQT1
							LQT2
							LQT3
							LQT4
							LQT5
							LQT7
Hummel et al., 2013	Observational	Y—SCN5a mutation 1795insD	Genotyped asymptomatic patients	NR	NR	LV end-diastolic dimension, LV mass, LA volume, EF, E, A-wave, E/A, DT, IVRT, e’	A
							LQT3
ter Bekke et al., 2015	Observational	Y—SM and DM—Hom and Het	Genotyped patients	NR	RR, QT, QT_C_	EF, EMW, QAoC	A and S
							LQT1
							LQT2
							LQT3
							LQT6
Robyns et al., 2017	Cross-sectional	Y	Patients with LQTS mutation and their genotype-negative family members who had both resting ECG and Holter recordings available were included	One patient with LQTS was excluded due to QT-RR correlation below 0.5	ECG RR, ECG QT_C_, ECG QT, Holter T-wave amplitude, Holter QT-RR slope, Holter mean QT, Holter number of templates, Holter RR	NR	NR
				Children younger than 8 years were excluded because of high heart rates			
Vink et al., 2018	Cross-sectional	Y	Children under 18 years old	Children above 18 years old	RR, QT_C_, QT	CD	NR
Charisopoulou et al., 2019	Observational—Retrospective Study	Y			RR, HR, QT_C_	EF, e’, a’, E/A, FT, ET, IVRT	LQT1
							LQT2

Abbreviations: Y, Yes; N, No; SM, Single-Mutation; DM, Double-Mutation; NR, not reported; QT_C_, corrected QT interval; RR, RR interval; EF, ejection fraction; CD, contraction duration; PEV, post-ejection velocity; A, asymptomatic; S, symptomatic; Hom, Homozygous; Het, Heterozygous; HR, Heart Rate; MD, mechanical dispersion; e’, early peak diastolic velocity; a’, late peak diastolic velocity; E, LV passive filling; DT, diastolic time; IVRT, interventricular relaxation time; EMW, electromechanical window; QAoC, interval from QRS onset to aortic-valve closure; FT, filling time; ET, ejection time; LVDd, left-ventricular diastolic diameter; and ThT, wall thickening time.

## Supplementary Materials

The following are available online at https://www.mdpi.com/2077-0383/9/9/2820/s1. Figure S1: Flow chart of study section, Figure S2: Electrical abnormalities in long-QT syndrome (LQTS) patients vs. control, Figure S3: Left ventricular (LV) systolic function difference: comparison between LQTS vs. control, Figure S4: LV diastolic function difference: comparison between LQTS vs. control, Figure S5: Electrical abnormalities in LQTS—comparison between symptomatic vs. asymptomatic, Figure S6: Electrical and mechanical abnormalities in LQT1 vs. LQT2, Table S1: Main characteristics of patients enrolled among trials included in the study, Table S2: Diagnostic accuracy of echocardiographic parameters in predicting CE in LQTS.
