# Supplementary material for: Left Ventricular Contraction Duration Is the Most Powerful Predictor of Cardiac Events in LQTS: A Systematic Review and Meta-Analysis
Source: J Clin Med. 2020 Aug 31;9(9):2820. doi: 10.3390/jcm9092820 (PMC7565502; doi:10.3390/jcm9092820)
Supplement: Supplementary file 1 [file jcm-09-02820-s001.pdf]

Supplementary data

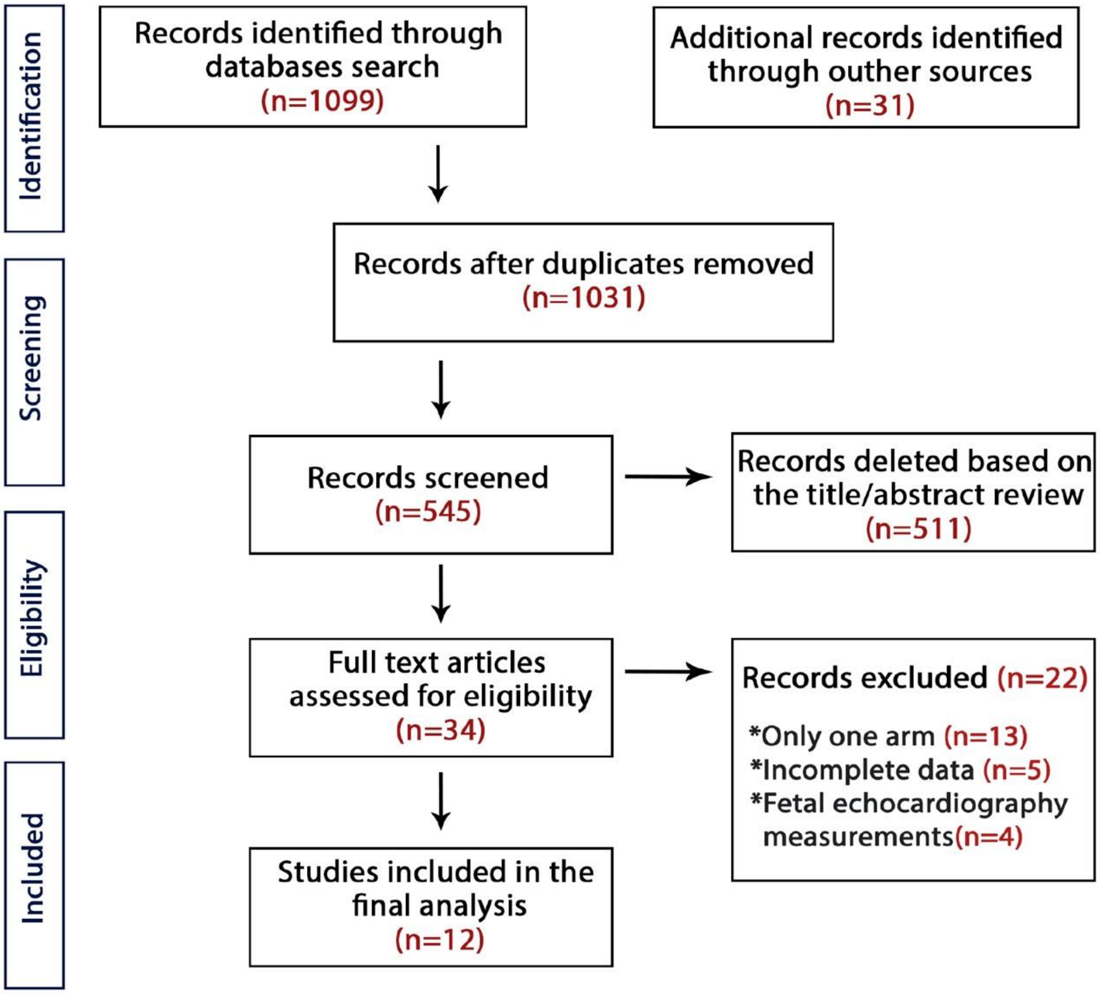

Figure S1. Flow chart of study section

a) QTc

Study or Subgroup	LQTS Mean	LQTS SD	LQTS Total	Control Mean	Control SD	Control Total	Weight	Mean Difference IV, Random, 95% CI	Year
Nakayama 1998	503	27	8	397	26	9	8.9%	106.00 [80.73, 131.27]	1998
Savoye 2003	454	37	10	363	29	14	8.3%	91.00 [63.49, 118.51]	2003
Haugaa 2009	477	35	91	390	20	20	12.9%	87.00 [75.66, 98.34]	2009
Hummel 2013	475	50	12	397	23	12	7.4%	78.00 [46.86, 109.14]	2013
Leren 2015	467	39	192	389	25	60	13.6%	78.00 [69.61, 86.39]	2015
Ter Bekke 2015	465	50	244	391	27	76	13.5%	74.00 [65.27, 82.73]	2015
Robyns 2016	447	56	69	376	27	55	11.9%	71.00 [55.98, 86.02]	2016
Vink 2018	431	39	41	387	40	41	11.2%	44.00 [26.90, 61.10]	2018
Charisopoulou 2019	453	42	47	413	17	35	12.4%	40.00 [26.74, 53.26]	2019
**Total (95% CI)**			**714**			**322**	**100.0%**	**72.81 [60.82, 84.81]**	

Heterogeneity: $Tau^2 = 256$; $Chi^2 = 50.43$, df = 8 ($P < 0.00001$); $I^2 = 64\%$

Test for overall effect: Z = 11.90 ($P < 0.00001$)

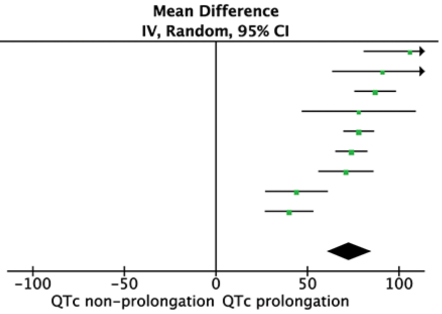

b) QTc dispersion

Study or Subgroup	LQTS Mean	LQTS SD	LQTS Total	Control Mean	Control SD	Control Total	Weight	Mean Difference IV, Random, 95% CI	Year
Priori 1994	73	26	28	48	12	15	41.7%	25.00 [13.61, 36.39]	1994
Nakayama 1998	81	39	8	20	8	10	25.2%	61.00 [33.52, 88.48]	1998
Hummel 2013	48	27	12	30	21	12	33.2%	18.00 [−1.35, 37.35]	2013
**Total (95% CI)**			**48**			**37**	**100.0%**	**31.74 [11.82, 51.65]**	

Heterogeneity: $Tau^2 = 213.9$; $Chi^2 = 6.82$, df = 2 ($P = 0.03$); $I^2 = 61\%$

Test for overall effect: Z = 3.12 ($P = 0.002$)

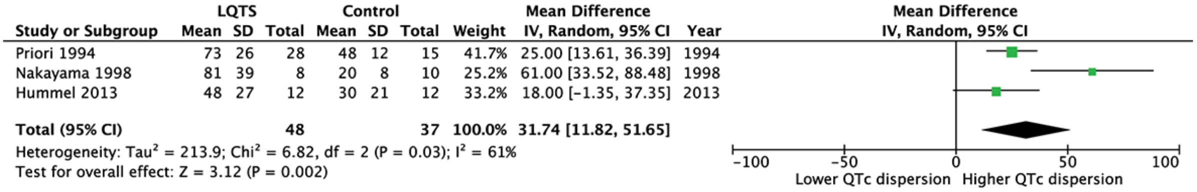

c) RR interval

Study or Subgroup	LQTS Mean	LQTS SD	LQTS Total	Control Mean	Control SD	Control Total	Weight	Mean Difference IV, Random, 95% CI	Year
Nakayama 1998	1,113	70	8	974	147	10	8.5%	139.00 [35.78, 242.22]	1998
Ter Bekke 2015	950	152	240	845	157	76	52.6%	105.00 [64.80, 145.20]	2015
Robyns 2016	953	167	69	901	157	55	26.9%	52.00 [−5.22, 109.22]	2016
Vink 2018	906	215	41	818	183	41	12.0%	88.00 [1.58, 174.42]	2018
**Total (95% CI)**			**358**			**182**	**100.0%**	**91.58 [61.39, 121.78]**	

Heterogeneity: $Tau^2 = 30.35$; $Chi^2 = 3.08$, df = 3 ($P = 0.38$); $I^2 = 3\%$

Test for overall effect: Z = 5.94 ($P < 0.00001$)

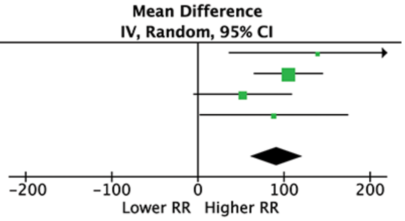

Figure S2. Electrical abnormalities in LQTS patients vs. control

LV systolic function

a) LVEF

Study or Subgroup	LQTS Mean	LQTS SD	LQTS Total	Control Mean	Control SD	Control Total	Weight	Mean Difference IV, Random, 95% CI	Year
Savoye 2003	69	7	10	70	8	14	1.9%	−1.00 [−7.03, 5.03]	2003
Hauguaa 2010	64	6	101	64	5	35	16.8%	0.00 [−2.03, 2.03]	2010
Hummel 2013	60	6	12	56	6	12	3.0%	4.00 [−0.80, 8.80]	2013
Leren 2015	61	5	192	61	5	60	32.9%	0.00 [−1.45, 1.45]	2015
Ter Bekke 2015	63	7	244	63	5	76	33.9%	0.00 [−1.43, 1.43]	2015
Charisopoulou 2019	72	5	47	70	6	35	11.5%	2.00 [−0.45, 4.45]	2019
**Total (95% CI)**			**606**			**232**	**100.0%**	**0.33 [−0.50, 1.16]**	

Heterogeneity: $Tau^2 = 0.00$; $Chi^2 = 4.73$, df = 5 (P = 0.45); $I^2 = 0\%$
Test for overall effect: Z = 0.78 (P = 0.43)

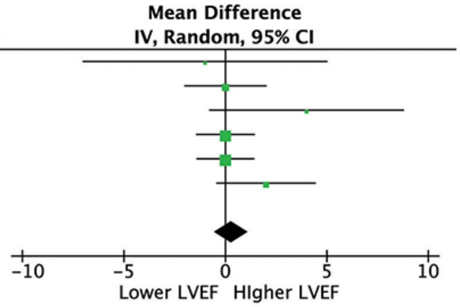

b) LV GLS

Study or Subgroup	LQTS Mean	LQTS SD	LQTS Total	Control Mean	Control SD	Control Total	Weight	Mean Difference IV, Fixed, 95% CI	Year
Hauguaa 2010	−21.4	1.8	101	−22.6	2	35	37.4%	1.20 [0.45, 1.95]	2010
Leren 2015	−22	2	192	−23	2	60	62.6%	1.00 [0.42, 1.58]	2015
**Total (95% CI)**			**293**			**95**	**100.0%**	**1.07 [0.62, 1.53]**	

Heterogeneity: $Chi^2 = 0.17$, df = 1 (P = 0.68); $I^2 = 0\%$
Test for overall effect: Z = 4.59 (P < 0.00001)

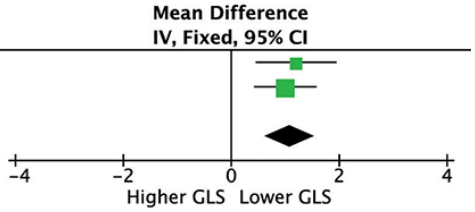

Figure S3. LV systolic function difference: comparison between LQTS vs. Control

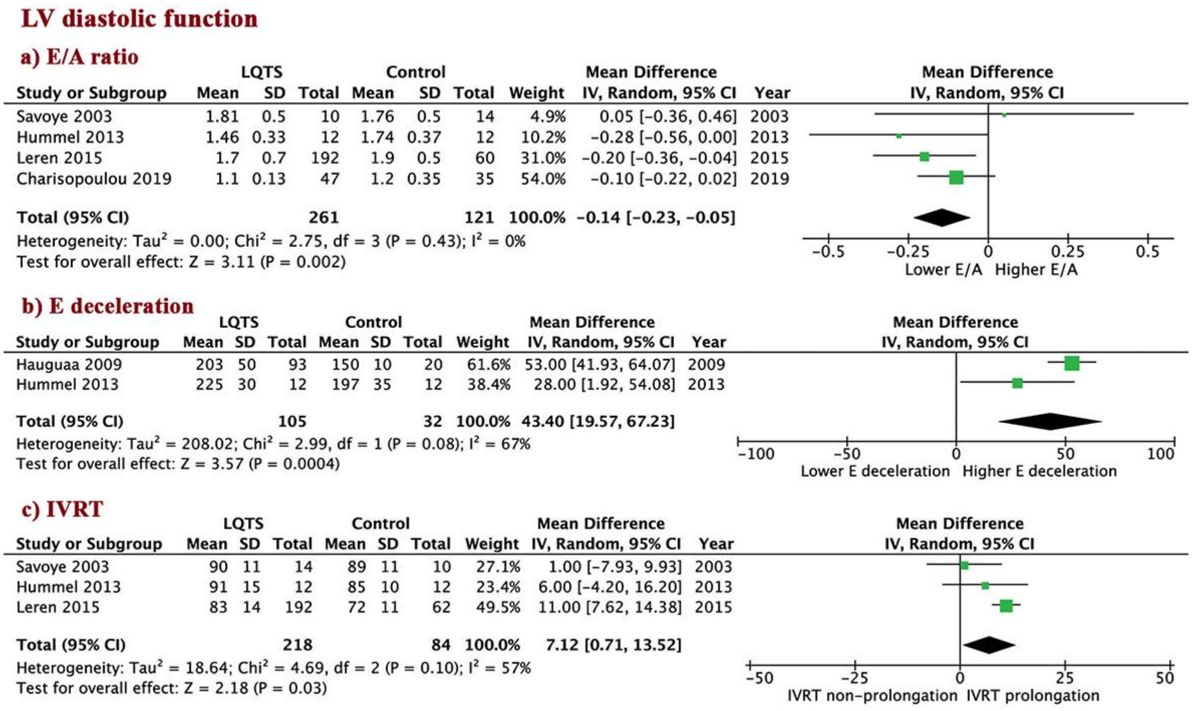

a) E/A ratio

Study or Subgroup	LQTS Mean	LQTS SD	LQTS Total	Control Mean	Control SD	Control Total	Weight	Mean Difference IV, Random, 95% CI	Year
Savoye 2003	1.81	0.5	10	1.76	0.5	14	4.9%	0.05 [−0.36, 0.46]	2003
Hummel 2013	1.46	0.33	12	1.74	0.37	12	10.2%	−0.28 [−0.56, 0.00]	2013
Leren 2015	1.7	0.7	192	1.9	0.5	60	31.0%	−0.20 [−0.36, −0.04]	2015
Charisopoulou 2019	1.1	0.13	47	1.2	0.35	35	54.0%	−0.10 [−0.22, 0.02]	2019
**Total (95% CI)**			**261**			**121**	**100.0%**	**−0.14 [−0.23, −0.05]**	

Heterogeneity: $Tau^2 = 0.00$; $Chi^2 = 2.75$, df = 3 (P = 0.43); $I^2 = 0\%$
Test for overall effect: Z = 3.11 (P = 0.002)

b) E deceleration

Study or Subgroup	LQTS Mean	LQTS SD	LQTS Total	Control Mean	Control SD	Control Total	Weight	Mean Difference IV, Random, 95% CI	Year
Hauguaa 2009	203	50	93	150	10	20	61.6%	53.00 [41.93, 64.07]	2009
Hummel 2013	225	30	12	197	35	12	38.4%	28.00 [1.92, 54.08]	2013
**Total (95% CI)**			**105**			**32**	**100.0%**	**43.40 [19.57, 67.23]**	

Heterogeneity: $Tau^2 = 208.02$; $Chi^2 = 2.99$, df = 1 (P = 0.08); $I^2 = 67\%$
Test for overall effect: Z = 3.57 (P = 0.0004)

c) IVRT

Study or Subgroup	LQTS Mean	LQTS SD	LQTS Total	Control Mean	Control SD	Control Total	Weight	Mean Difference IV, Random, 95% CI	Year
Savoye 2003	90	11	14	89	11	10	27.1%	1.00 [−7.93, 9.93]	2003
Hummel 2013	91	15	12	85	10	12	23.4%	6.00 [−4.20, 16.20]	2013
Leren 2015	83	14	192	72	11	62	49.5%	11.00 [7.62, 14.38]	2015
**Total (95% CI)**			**218**			**84**	**100.0%**	**7.12 [0.71, 13.52]**	

Heterogeneity: $Tau^2 = 18.64$; $Chi^2 = 4.69$, df = 2 (P = 0.10); $I^2 = 57\%$
Test for overall effect: Z = 2.18 (P = 0.03)

Figure S4. LV diastolic function difference: comparison between LQTS vs. Control

Electrical abnormalities in LQTS

a) QTc

Study or Subgroup	LQTS Symptomatic Mean	SD	Total	LQTS Asymtomatic Mean	SD	Total	Weight	Mean Difference IV, Random, 95% CI	Year
Nakayama 1998	511	13	6	503	13	2	10.1%	8.00 [−12.80, 28.80]	1998
Hauguaa 2009	510	45	42	460	30	31	13.0%	50.00 [32.77, 67.23]	2009
Hauguaa 2010	495	50	48	460	30	53	13.9%	35.00 [18.71, 51.29]	2010
Hauguaa 2013	498	60	91	464	36	125	16.7%	34.00 [20.15, 47.85]	2013
Leren 2015	486	47	69	456	29	123	18.9%	30.00 [17.78, 42.22]	2015
Ter Bekke 2015	488	50	97	450	44	147	18.8%	38.00 [25.77, 50.23]	2015
Charisopoulou 2019	497	43	20	457	36	27	8.6%	40.00 [16.77, 63.23]	2019
**Total (95% CI)**			**373**			**508**	**100.0%**	**34.11 [26.26, 41.95]**	

Heterogeneity: $Tau^2 = 46.07$; $Chi^2 = 10.40$, df = 6 (P = 0.11); $I^2 = 42\%$

Test for overall effect: Z = 8.52 (P < 0.00001)

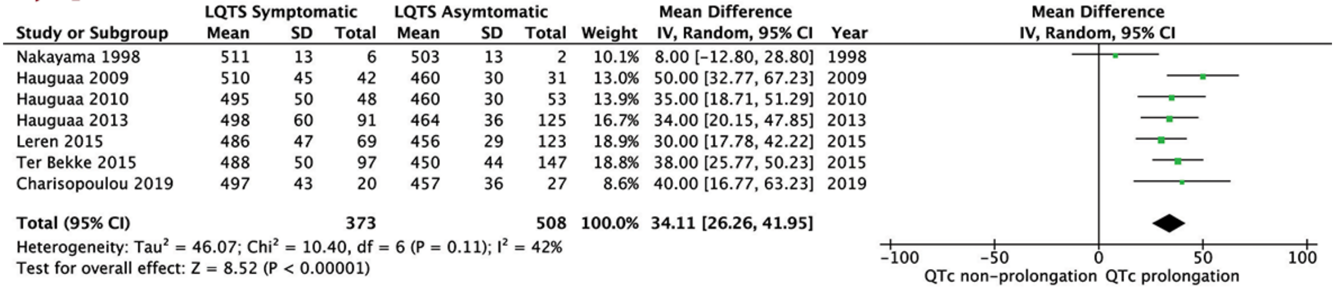

Figure S5. Electrical abnormalities in LQTS: comparison between symptomatic vs. asymptomatic

a) QTc

Study or Subgroup	LQT1 Mean	LQT1 SD	LQT1 Total	LQT2 Mean	LQT2 SD	LQT2 Total	Weight	Mean Difference IV, Random, 95% CI	Year
Hauguaa 2010	470	35	64	470	30	26	14.3%	0.00 [−14.37, 14.37]	2010
Leren 2015	462	34	130	463	30	53	29.8%	−1.00 [−10.97, 8.97]	2015
Ter Bekke 2015	491	54	38	489	50	42	5.7%	2.00 [−20.88, 24.88]	2015
Charisopoulou 2019	458	15	36	451	10	11	50.2%	7.00 [−0.68, 14.68]	2019
**Total (95% CI)**			**268**			**132**	**100.0%**	**3.33 [−2.11, 8.77]**	

Heterogeneity: $Tau^2 = 0.00$; $Chi^2 = 1.82$, df = 3 (P = 0.61); $I^2 = 0\%$
Test for overall effect: Z = 1.20 (P = 0.23)

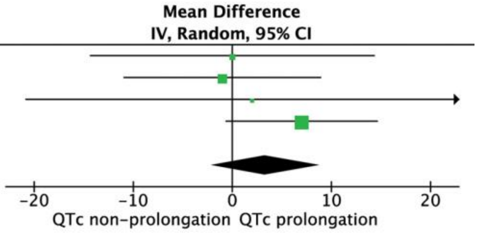

b) Contraction duration

Study or Subgroup	LQT1 Mean	LQT1 SD	LQT1 Total	LQT2 Mean	LQT2 SD	LQT2 Total	Weight	Mean Difference IV, Random, 95% CI	Year
Hauguaa 2010	441	63	130	461	62	53	45.8%	−20.00 [−39.90, −0.10]	2010
Leren 2015	425	37	130	418	41	53	54.2%	7.00 [−5.74, 19.74]	2015
**Total (95% CI)**			**260**			**106**	**100.0%**	**−5.37 [−31.74, 20.99]**	

Heterogeneity: $Tau^2 = 291.85$; $Chi^2 = 5.02$, df = 1 (P = 0.03); $I^2 = 70\%$
Test for overall effect: Z = 0.40 (P = 0.69)

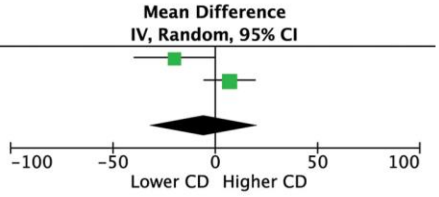

c) Mechanical dispersion

Study or Subgroup	LQT1 Mean	LQT1 SD	LQT1 Total	LQT2 Mean	LQT2 SD	LQT2 Total	Weight	Mean Difference IV, Random, 95% CI	Year
Hauguaa 2010	31	14	64	37	14	26	38.8%	−6.00 [−12.38, 0.38]	2010
Leren 2015	31	13	130	32	14	53	61.2%	−1.00 [−5.38, 3.38]	2015
**Total (95% CI)**			**194**			**79**	**100.0%**	**−2.94 [−7.71, 1.84]**	

Heterogeneity: $Tau^2 = 4.70$; $Chi^2 = 1.60$, df = 1 (P = 0.21); $I^2 = 38\%$
Test for overall effect: Z = 1.21 (P = 0.23)

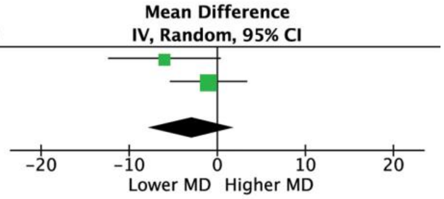

d) QAoC

Study or Subgroup	LQT1 Mean	LQT1 SD	LQT1 Total	LQT2 Mean	LQT2 SD	LQT2 Total	Weight	Mean Difference IV, Random, 95% CI	Year
Ter Bekke 2015	420	50	38	413	31	42	78.4%	7.00 [−11.46, 25.46]	2015
Charisopoulou 2019	390	45	36	385	54	11	21.6%	5.00 [−30.13, 40.13]	2019
**Total (95% CI)**			**74**			**53**	**100.0%**	**6.57 [−9.77, 22.91]**	

Heterogeneity: $Tau^2 = 0.00$; $Chi^2 = 0.01$, df = 1 (P = 0.92); $I^2 = 0\%$
Test for overall effect: Z = 0.79 (P = 0.43)

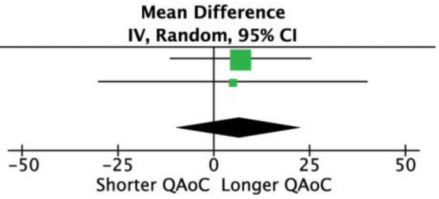

e) EMW

Study or Subgroup	LQT1 Mean	LQT1 SD	LQT1 Total	LQT2 Mean	LQT2 SD	LQT2 Total	Weight	Mean Difference IV, Random, 95% CI	Year
Ter Bekke 2015	−68	37	38	−68	43	42	35.5%	0.00 [−17.54, 17.54]	2015
Charisopoulou 2019	−40	23	36	−48	18	11	64.5%	8.00 [−5.02, 21.02]	2019
**Total (95% CI)**			**74**			**53**	**100.0%**	**5.16 [−5.30, 15.61]**	

Heterogeneity: $Tau^2 = 0.00$; $Chi^2 = 0.52$, df = 1 (P = 0.47); $I^2 = 0\%$
Test for overall effect: Z = 0.97 (P = 0.33)

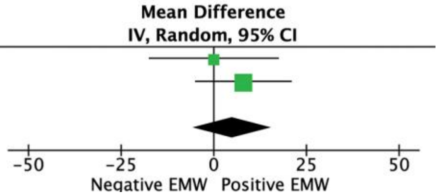

Figure S6. Electrical and mechanical abnormalities in LQT1 vs. LQT2

Table S1. Main characteristics of patients enrolled among trials included in the study

Study (year)	Arms	Sample Size	Age (year)	Female (%)	QTc (ms)	HR (bpm)	LVEF (%)
Priori et al., 1994	P	28	17.3 ± 5.6	53.3	75	519 ± 41	NR
	C	15	17 ± 4	53.3	53	418 ± 22	NR
	S	18	17 ± 4.5	77.7	NR	NR	NR
	A	10	17 ± 8	80	NR	NR	NR
Nakayama et al., 1998	P	8	27 ± 17	50	509 ± 27	NR	NR
	C	10	23 ± 6	40	397 ± 26	NR	NR
	S	6	19.5 ± 7.48	50	512 ± 13	NR	NR
	A	2	29.5 ± 6.5	50	503 ± 3	NR	NR
Savoye et al., 2003	P	10	35.7 ± 12	70	454 ± 37	NR	69 ± 7
	C	14	33.6 ± 9	50	363 ± 29	NR	70 ± 8
	S	NR	NR	NR	NR	NR	NR
	A	NR	NR	NR	NR	NR	NR
Leren et al., 2015	P	192	36 ± 16	61	467 ± 39	64 ± 12	61 ± 5
	C	60	37 ± 10	53	389 ± 25	65 ± 10	61 ± 5
	S	69	32 ± 16	74	486 ± 47	62 ± 11	61 ± 6
	A	123	38 ± 16	54	456 ± 29	65 ± 12	60 ± 5
Haugaa et al., 2009	P	73	NR	NR	NR	NR	NR
	C	20	34 ± 11	55	390 ± 20	NR	67 ± 3
	S	42	32 ± 16	79	497 ± 42	NR	64 ± 6
	A	31	41 ± 14	68	460 ± 30	NR	64 ± 6
Haugaa et al., 2010	P	101	37 ± 16	70	NR	65 ± 13	64 ± 6
	C	35	34 ± 10	57	NR	69 ± 10	64 ± 5
	S	48	32 ± 16	81	495 ± 50	64 ± 13	64 ± 5
	A	53	41 ± 14	60	460 ± 30	67 ± 13	64 ± 6
Haugaa et al., 2013	P	216	23 ± 17	59	478 ± 50	70 ± 17	NR
	C	NR	NR	NR	NR	NR	NR

	S	91	24 ± 16	66	498 ± 60	65 ± 15	NR
	A	125	23 ± 17	50	464 ± 36	73 ± 17	NR
Hummel et al., 2013	P	12	35.7 ± 7.3	75	475 ± 50	64 ± 11	60 ± 6
	C	12	35.3 ± 6.2	58	397 ± 23	68 ± 11	56 ± 5
	S	NR	NR	NR	NR	NR	NR
	A	NR	NR	NR	NR	NR	NR
ter Bekke et al., 2015	P	244	38 ± 16	67	465 ± 50	NR	63 ± 5
	C	76	37 ± 12	67	391 ± 27	NR	63 ± 7
	S	97	37 ± 17	73	488 ± 50	NR	NR
	A	147	39 ± 16	63	450 ± 44	NR	NR
Robyns et al., 2017	P	69	34 ± 17	54	447 ± 56	NR	NR
	C	55	35 ± 16	44	376 ± 27	NR	NR
	S	19	NR	NR	NR	NR	NR
	A	50	NR	NR	NR	NR	NR
Vink et al., 2018	P	41	NR	37	431 ± 30	NR	NR
	C	41	NR	37	387 ± 17	NR	NR
	S	5	NR	NR	NR	NR	NR
	A	36	NR	NR	NR	NR	NR
Charisopoulou et al., 2019	P	47	45 ± 15	53	477.0 ± 40.0	86.3 ± 12.6	71.0 ± 4.11
	C	35	47 ± 13	54	405.7 ± 17.9	85.3 ± 11.5	73.3 ± 5.51
	S	20	NR	NR	495.9 ± 39.2	87.3 ± 13.0	NR
	A	27	NR	NR	468.3 ± 16.3	92.7 ± 16.5	NR

Abbreviations: P: Patients with LQTS; C: Control; S: Symptomatic; A: Asymptomatic; NR: Not rep

Table S2. Diagnostic accuracy of echocardiographic parameters in predicting CE in LQTS

	**Sensitivity**	**Specificity**	**PPV**	**NPV**	**Accuracy**	**DOR**
CD≥430ms	71 [40-94]	84 [67-94]	75 [62 – 84]	87 [72 – 92]	83 [73 – 90]	>19.5
EMW -59ms	82 [66-93]	56 [41-70]	56 [51 – 61]	83 [77 – 88]	67 [61 – 71]	>7.47
QTc≥460ms	53 [37-75]	73 [60-82]	59 [53 – 63]	75 [70 – 78]	68 [62 – 71]	>4.14

CD: Contraction duration; EMW: electromechanical window; QTc: QT corrected.
